# Morpheus: a user-friendly modeling environment for multiscale and multicellular systems biology

**DOI:** 10.1093/bioinformatics/btt772

**Published:** 2014-01-17

**Authors:** Jörn Starruß, Walter de Back, Lutz Brusch, Andreas Deutsch

**Affiliations:** Center for Information Services and High Performance Computing, Technische Universität Dresden, 01062 Dresden, Germany

## Abstract

**Summary:** Morpheus is a modeling environment for the simulation and integration of cell-based models with ordinary differential equations and reaction-diffusion systems. It allows rapid development of multiscale models in biological terms and mathematical expressions rather than programming code. Its graphical user interface supports the entire workflow from model construction and simulation to visualization, archiving and batch processing.

**Availability and implementation:** Binary packages are available at http://imc.zih.tu-dresden.de/wiki/morpheus for Linux, Mac OSX and MS Windows.

**Contact:** walter.deback@tu-dresden.de

**Supplementary information:** Supplementary data are available at *Bioinformatics* online.

## 1 INTRODUCTION

Systems biology is rapidly expanding its scope from the study of intracellular networks to the analysis of tissue- and organ-scale systems. This increases the need for computational methods and software that support the simulation and integration of cell-based models with models for intra- and extracellular dynamics. Currently available modeling environments typically require programming or scripting to account for dynamical feedback between submodels at different scales (Andasari *et al.*, 2012; Mirams *et al.*, 2013; Swat *et al.*, 2012). Yet, as models grow more complex, it becomes increasingly important to separate modeling from implementation, to automate multiscale model integration and to provide intuitive user interfaces (Sütterlin *et al.*, 2013).

Morpheus is a user-friendly application for the modeling, simulation and integration of cell-based models, ordinary differential equations and reaction-diffusion systems. It supports the construction of multiscale models using biological terminology and mathematical constructs, based on a novel domain-specific language. Its graphical user interface (GUI) supports the whole workflow from model development and simulation to visualization and data output and features tools for archiving and batch processing.

**Fig. 1.**
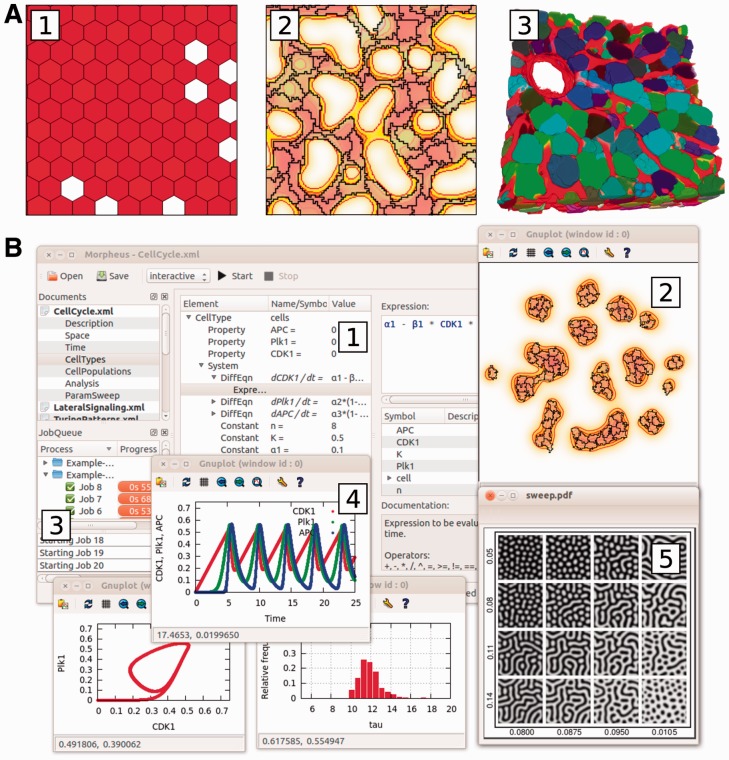
(**A**) Morpheus supports the simulation and integration of diverse modeling approaches including (1) cell-based, (2) multiscale and (3) image-based modeling, see case studies in main text. (**B**) The user interface provides workflow tools for (1) model editing, (2) visualization, (3) archiving, (4) data analysis and (5) batch processing

Morpheus has hitherto been used in a variety of studies, including collective motion (Starruß *et al.*, 2007), morphogenesis (Köhn-Luque *et al.*, 2011, 2013) and cell fate decisions (de Back *et al.*, 2013a, b) (Fig. 1A).

## 2 SOFTWARE

### 2.1 Model formalisms

Modeling of multicellular systems requires methods that go beyond the standard repertoire of model formalisms in systems biology (Machado *et al.*, 2011). Cells must typically be modeled as discrete interacting entities, sometimes as motile objects, possibly even with articulated 3D cell shapes. Morpheus provides methods to define cell-based models of interactions between discrete cells, spatially represented as point-like objects or with explicit 2D/3D cell shapes. Cell motility, adhesion and biophysical constraints can be added using the cellular Potts model (CPM) framework (Graner *et al.*, 1992).

Cells can be linked to models of intracellular biological pathways such as gene regulatory networks and signaling pathways. These can be specified in the form of ordinary, stochastic and delay differential equations or imported from SBML-based models. Morpheus also supports the simulation of reaction-diffusion systems. This can be used to couple cellular behavior to morphogen concentrations in the extracellular environment.

Apart from these core formalisms, a range of auxiliary models can be constructed including coupled lattice ODEs, finite state machines, gradient-based models and cellular automata. All formalisms can be used as single-scale models or integrated into multiscale models. Example use cases are described on the Web site and are available in the GUI.

### 2.2 Model description

Morpheus separates modeling from numerical implementation by using a declarative domain-specific markup language. The model description language allows users to describe their models in biological and mathematical terms. Mathematical expressions such as functions, equations, events and systems of differential equations can be specified in common infix notation to describe the interactions and dynamics of cell-bound variables and molecular species in reaction-diffusion systems, based on symbolic identifiers.

Multiscale interactions are described by linking symbolic identifiers associated with different spatiotemporal models. Internally, Morpheus automatically integrates these models by appropriately mapping data between spatial models and scheduling numerical updates according to the dependencies between symbols.

Model descriptions also specify aspects related to the simulation of the model, such as time and spatial discretization, initial and boundary conditions as well as data output and visualization. The full model specification is stored in a single file in XML format, aiding archiving and model exchange. The same format is used to periodically store the simulation state during execution.

The model description language and model integration is described in more detail in the Supplementary Information and on the Web site.

### 2.3 Graphical user interface

GUI (Fig. 1B) assists the user in the workflow from model development to data analysis. The model editor supports model construction by, for example, copy/pasting between models, disabling/enabling model components and providing context-sensitive documentation. It provides a job scheduler that supports multithreaded and parallel job execution. For parameter exploration and sensitivity analysis, ranges of values can be specified for batch processing of any set of model parameters. Simulation results are archived with their associated models in a folder structure that can be browsed from within the GUI. Archived models can be restored and resumed for further analysis.

Output data can be analyzed at runtime, written to plain text files for *post hoc* analysis and visualized using the flexible gnuplot interface. For 3D data, the multichannel TIFF format is supported, providing an interface to image analysis software such as ImageJ.

## 3 CASE STUDIES

To date, the Morpheus modeling and simulation environment has been used in a diverse range of studies including the study of collective motion in *Myxococcus xantus* using an extension of the CPM (Starruß *et al.*, 2007), the investigation of transdifferentiation and pattern formation in the pancreas using a coupled ODE lattice model (de Back *et al.*, 2013a, b) (Fig. 1A1) and modeling of vascular morphogenesis using a coupled CPM/reaction-diffusion model (Köhn-Luque *et al.*, 2011, 2013; Fig. 1A2). Currently, the software is also being used for image-based modeling by integration of spatial information from microscopy images into simulation models (e.g. liver tissue, Fig. 1A3). Morpheus has also proven to be useful in the classroom, for students in mathematics and physics as well as in biology.

## Supplementary Material

Supplementary Data
