# Supplementary material for: Morpheus: a user-friendly modeling environment for multiscale and multicellular systems biology
Source: Bioinformatics. 2014 Jan 17;30(9):1331–2. doi: 10.1093/bioinformatics/btt772 (PMC3998129; doi:10.1093/bioinformatics/btt772)
Supplement: Supplementary Data [file supp_30_9_1331__index.html]

Morpheus: a user-friendly modeling environment for multiscale and multicellular systems biology — Supplementary Data 

# Morpheus: a user-friendly modeling environment for multiscale and multicellular systems biology

## Supplementary Data

files

**Files in this Data Supplement:**

- Supplementary Data - pdf file
