# Supplementary material for: Morpheus: a user-friendly modeling environment for multiscale and multicellular systems biology
Source: Bioinformatics. 2014 Jan 17;30(9):1331–2. doi: 10.1093/bioinformatics/btt772 (PMC3998129; doi:10.1093/bioinformatics/btt772)
Supplement: Supplementary Data [file supp_btt772_Morpheus_Supplement.pdf]

Morpheus

User manual

Walter de Back and Jörn Starruß

Center for Information Services and High Performance Computing
Technische Universität Dresden, 01062 Dresden, Germany
http://imc.zih.tu-dresden.de/wiki/morpheus

Disclaimer

Non-commercial use: Morpheus (the Software) is distributed for academic use and cannot be used for commercial gain without explicitly written agreement by the Developers. No warranty: The Software is provided "as is" without warranty of any kind, either express or implied, including without limitation any implied warranties of condition, uninterrupted use, merchantability, fitness for a particular purpose, or non-infringement. No liability: The Developers do not accept any liability for any direct, indirect, incidential, special, exemplary or consequential damages arising in any way out of the use of the Software.

This manual describes Morpheus version 1.1.0 (revision 866).

Development of Morpheus was supported by the German Ministry for Education and Research (BMBF) through grants 0315734 and 0316169.

Department for Innovative Methods of Computing
Center for Information Services and High Performance Computing
Technische Universität Dresden, 01062 Dresden, Germany
http://imc.zih.tu-dresden.de/imc/

Contents

Contents **i**

1 Introduction **1**
1.1 About Morpheus . . . 1
1.1.1 Software . . . 1
1.1.2 Usability . . . 2
1.1.3 Developers . . . 3
1.2 About this manual . . . 3
1.2.1 Margin images . . . 3
1.2.2 More information . . . 3

2 Graphical user interface **5**
2.1 Model construction and editing . . . 5
2.1.1 Examples . . . 6
2.1.2 Documents panel . . . 6
2.1.3 Element editor . . . 6
2.1.4 Attribute editor . . . 6
2.1.5 Expression editor . . . 6
2.1.6 Documentation . . . 7
2.1.7 Clipboard . . . 7
2.1.8 Fixboard . . . 7
2.2 Simulation and results . . . 7
2.2.1 Simulation execution . . . 7
2.2.2 Status and error messages . . . 8
2.2.3 Job queue and archive . . . 8
2.2.4 Simulation output . . . 8
2.2.5 Restoring and checkpointing . . . 10
2.2.6 SBML import . . . 10
2.3 Parameter sweeps . . . 11
2.3.1 Multiple parameters . . . 11
2.3.2 Simulation and results . . . 13
2.3.3 Restoring parameter sweeps . . . 13

3 Model formalisms **15**
3.1 The `System` construct . . . 15
3.2 Differential equations . . . 15
3.2.1 Finite difference solvers . . . 16
3.2.2 Stochastic differential equations . . . 16
3.2.3 Delay differential equations . . . 17
3.2.4 Initial condition . . . 17
3.3 Reaction-diffusion models . . . 17
3.3.1 Diffusion . . . 18
3.3.2 Boundary conditions . . . 18
3.3.3 Initial condition . . . 18
3.4 Cellular Potts model . . . 19
3.4.1 Modified Metropolis kinetics . . . 19
3.4.2 Stepper . . . 20
3.4.3 Neighborhood . . . 20
3.4.4 Monte Carlo Step (MCS) and time scaling . . . 20
3.4.5 Cell shape, interaction and motility . . . 21
3.4.6 Initial condition of spatial configuration . . . 22
3.5 Auxiliary model formalisms . . . 23

4 Model description language **25**
4.1 Declarative . . . 25
4.2 Domain-specific . . . 25
4.3 Encapsulation . . . 26
4.4 Two-tiered architecture . . . 26
4.4.1 XML . . . 26
4.4.2 Symbols . . . 27
4.5 Mathematical constructs . . . 28
4.6 XML schema . . . 28

5 Model integration **33**
5.1 Spatial mapping . . . 33
5.1.1 Automated mapping . . . 34
5.1.2 Explicit mapping with `Reporters` . . . 34
5.2 Scheduling . . . 34
5.2.1 Simulation schedule . . . 34
5.2.2 Scheduling update order . . . 36
5.2.3 Update interval . . . 36

1

Introduction

1.1 About Morpheus

Morpheus is a modeling and simulation environment for the study of multiscale multicellular systems. It supports the simulation of discrete cell-based models as well as ordinary differential equations and reaction-diffusion systems and facilitates the integration of these models into multiscale models. It allows users to construct and simulate models of gene regulation, signaling pathways, tissue patterning and morphogenesis and explore the effects of multiscale feedbacks between these processes.

1.1.1 Software

Morpheus is a self-contained application that covers the workflow from model construction to simulation and data analysis. Internally, it consists of two stand-alone components: a graphical user interface (`morpheus-gui`) and a simulator (`morpheus`). The graphical user interface provides tools for model editing, job execution and archiving and it generates XML-based model descriptions based on user-specified models. The simulator takes these model description files as input to perform numerical simulation and generate data output and visualization. The simulator can also be used from the command line interface.

1.1.1.1 Download and installation

Morpheus is available as binary package for all major operating systems. These can be downloaded from the download page of the Morpheus website.

For Linux, Morpheus is available as a Debian package (`.deb`) that can be installed using the default package manager. For other Linux distributions, these packages can be converted using tools such as `alien`.

For Mac OSX 10.6 or higher, Morpheus is distributed as an Apple disk image (`.dmg`) containing the application bundle (`.app`). Installing `Morpheus.app` is done by dragging the app into Applications, as usual. It also contains a `Gnuplot.app` required for data visualization, to be installed in the same way.

For MS Windows XP or higher, Morpheus uses an installer that guides the user through the installation process. Gnuplot for windows should be installed separately for which a recent version can be downloaded from the gnuplot sourceforge website.

1.1.1.2 External software

Morpheus is implemented in C++ and Qt and depends on a number of external libraries and software packages. The simulator (morpheus) is implemented in C++. It uses XMLParser to read model description files and write simulation states (checkpointing). Pseudo-random numbers are generated using Mersenne Twister 19937 from the C++11 standard library. Mathematical expressions are parsed and evaluated using the math parser library muparser. TIFF image stacks are read and written using libTIFF. For data visualization, it depends on the graphing utility Gnuplot and its C interface. Morpheus uses openMP for shared-memory parallel processing.

The graphical user interface (morpheus-gui) is implemented in the cross-platform framework Qt/C++. It uses a SQLite database to store an archive of jobs and parameter sweeps. It depends on libSSH for secure communication to remote high performance computing resources for remote job submission (ssh) and synchronization of simulation results (sftp). Furthermore, it depends on libSBML to import biochemical network models written in SBML format.

1.1.2 Usability

Improving usability and transparency of computational tools in multicellular systems biology is a prime objective in the design of Morpheus. This widens the audience from computational experts to more biologically trained students and researchers within this multi-disciplinary field and it can accelerate research by facilitating rapid development of new hypotheses into computational models and simulation results. Ultimately, it is required to streamline the scientific workflow in the face of the growing complexity of computational models in systems biology.

The usability and workflow management in Morpheus is most tangible in the graphical user interface with its tools for model construction, simulation, batch processing and archiving. However, it also extends into the modular design of the model formalisms, generating the flexibility to construct a range of auxiliary model formalisms. Moreover, the domain-specific language that Morpheus uses for model description separates the process of modeling from its numerical implementation and allows users to construct complex models in a transparent fashion.

1.1.2.1 Target audience

Morpheus is aimed at researchers and students interested in mathematical modeling of multicellular systems. Its use does not presuppose knowledge of computational modeling or programming. It is suitable for anyone with basic knowledge of mathematical modeling of biological systems including researchers and students with a background in biology, mathematics, physics and related fields.

1.1.2.2 Limitations

Morpheus is not an advanced solver for ordinary differential equations or reaction-diffusion systems. The use of explicit forward solver methods precludes the sim-

ulation of stiff systems, and the use of fixed time stepping methods requires the specification of reasonable time-steps by the user to ensure numerical stability under acceptable computational performance. The current version does not support solving partial differential equations with spatial derivatives other than the most commonly used second order and thereby excludes the simulation of e.g. advective terms.

1.1.3 Developers

Morpheus has been developed by Jörn Starruß, Walter de Back and others in the group of Prof. Andreas Deutsch at the Center for Information Services and High Performance Computing (ZIH) at the Technische Universität Dresden.

1.2 About this manual

This manual provides information on the main functionalities of the graphical user interface (chapter 2), the core model formalisms it implements (chapter 3), the domain-specific language in which Morpheus models are expressed (chapter 4) and the methods for model integration of the various formalisms (chapter 5).

The manual does not provide an extensive list of descriptions of features or model components. For documentation on individual model elements, users are refered to the Documentation panel in the graphical user interface (see section 2.1.6 and figure 2.1).

It intends to concisely describe Morpheus to a degree that is relevant for constructing models, performing simulation experiments and understanding how simulation results are achieved.

1.2.1 Margin images

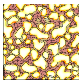

Images in the page margins refer to example models that are associated with the text and contain hyperlinks to the example website. The example models illustrate the main modeling features of Morpheus, introduce the model description language, and can be used as templates for new models. These example models are embedded in the application (see 2.1.1) and are described on the website (see figure 5.1).

1.2.2 More information

1.2.2.1 Documentation in GUI

Further documentation on individual elements of a Morpheus model is available in the graphical user interface in the Documentation panel (see section 2.1.6 and figure 2.1).

1.2.2.2 Website

The Morpheus website provides additional up-to-date information. It presents a growing number of example models that represent common use cases. The website also answers frequently asked questions, mentions how to submit bug reports, presents a comparison to related software and a detailed performance analysis.

Published articles on Morpheus and an up-to-date list of research papers in which the software has been used are listed on the publication page. When publishing results that were obtained with the help of Morpheus, please cite the article on Morpheus as indicated on the publication page. This helps to maintain Morpheus and support its further development.

The URL of the website is: `http://imc.zih.tu-dresden.de/wiki/morpheus`.

2

Graphical user interface

The graphical user interface (GUI) provides tools to construct and edit models, execute simulations, browse results and perform parameter sweeps.

2.1 Model construction and editing

The GUI provides a series of editing tools that facilitate the construction and modification of computational models and performs validation of the generated XML models (see figure 2.1).

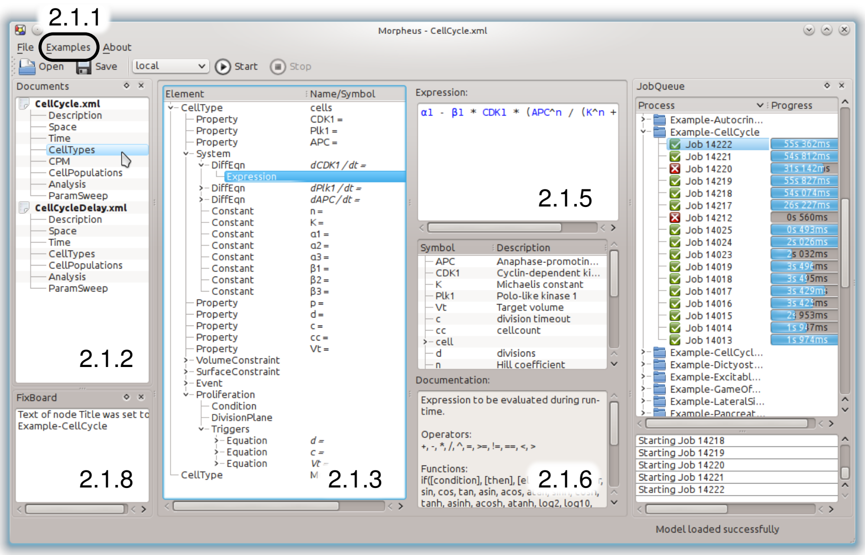

Figure 2.1: **Editor view**. This view is opened when selecting an element in the Documents panel. Numbers correspond to sections in the text.

2.1.1 Examples

A number of example models is included in the application, accessible through the Examples menu in main toolbar. These examples demonstrate key features of Morpheus, show diverse use cases and modeling approaches and illustrate all elements of the Morpheus model description language.

These example models can serve as templates for the construction of new models. Detailed descriptions can be found on the website (fig. 5.1).

2.1.2 Documents panel

The Documents panel gives an overview of the opened documents and provides a means to switch the current model. The main elements of each model are shown and can be edited via the context menu (right-click or ctrl-click). The context menu also allows the user to view the generated XML document.

2.1.3 Element editor

The Editor panel shows the editable tree-like structure of elements of the currently selected element in the Documents view. Elements can be added, copied or removed using the context menu. Adding an element opens a window showing a list of elements that can be added in the selected parent element. Required elements cannot be cut or removed, as to ensure model validity.

An element can also be temporarily disabled, which renders it ineffective without removing it from the model. Disabled elements can afterwards be re-enabled.

2.1.4 Attribute editor

The Attribute panel shows a table of attributes of the element that is currently selected in the Editor panel. The first column shows attribute names and, for optional attributes, a checkbox. The second column allows users to specify parameter values, depending on the type of attribute (double, integer, vector, string, etc.). Entries are validated by regular expressions. As long as an entry is invalid, it is marked by a red background.

2.1.5 Expression editor

The Expression panel is a multiline editor to enter or edit the expression that is currently selected in the Editor panel. The editor enables users to specify mathematical expressions as text in a familiar infix notation, using common operators and functions (listed in table 4.4). Expressions are formulated using predefined and user-defined symbols (see table 4.1). For each model, the available symbols are shown in a list below the editor.

2.1.6 Documentation

The Documentation panel provides a context-sensitive documentation of the element or attribute that is currently selected in the Editor or Attribute editor.

2.1.7 Clipboard

The Clipboard provides the ability to cut, copy and paste model elements. Because the clipboard is shared between documents, elements can be copied and pasted between models.

2.1.8 Fixboard

When loading documents with outdated or broken (yet well-formed) models, Morpheus attempts to repair the model by e.g. adding required elements or removing elements that are not allowed. The Fixboard provides a list of changes that have been made. Items in this list link to the element to which the changes have been applied.

2.2 Simulation and results

The GUI provides tools to execute (multiple) simulations called jobs, view simulation results, browse and restore jobs in the job archive and perform parameter sweeps (see figure 2.2).

2.2.1 Simulation execution

Simulations are executed using the Start button in the simulation toolbar. Jobs can be executed in interactive, local and remote mode.

Interactive mode is the most verbose mode and useful for model testing. Graphical output generated by Gnuplot is displayed on screen (overriding the settings in analysis plugins). And error messages are displayed in a pop-up window. The Stop button in the toolbar terminates the most recently started running interactive simulation.

Local mode is the standard, non-verbose, execution mode for simulation on a local machine. Graphical output is stored in files. Error messages are displayed below the job queue. The Stop button in the toolbar is disabled.

Remote mode is th execution mode for large-scale simulation or batch processing. Jobs are executed on a remote high performance computing resource via ssh, using the remote batch system (currently, only LSF is supported). See File/Settings/Remote.

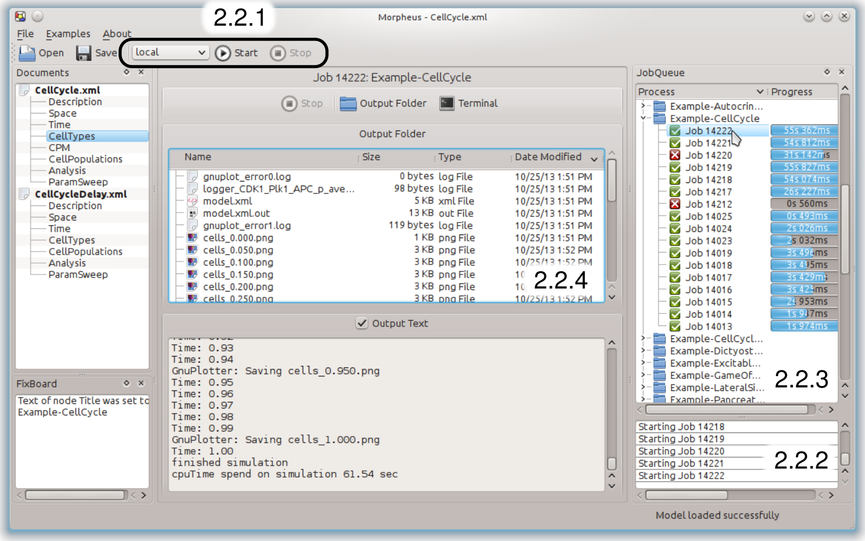

Figure 2.2: **Results view**. This view is opened at simulation start or upon selection a job in the job archive (JobQueue). Numbers correspond to sections in the text.

2.2.2 Status and error messages

Status and error messages of simulations executed in local mode are non-intrusively displayed in the message box below the JobQueue panel. In interactive mode, these error messages are displayed in a pop-up window.

2.2.3 Job queue and archive

The JobQueue panel provides an overview of pending, running, and completed simulation jobs and shows the progress and execution time. Jobs can be stopped, removed and debugged (requires GNU debugger `gdb`) using the context menu. Jobs can be grouped and sorted according to model, job ID, state or sweep.

The result browser panel displays the content of the output folder of the currently selected job. The toolbar allows jobs to be stopped (when running) and to open the output folder in a system-dependent file browser, or a command line terminal.

2.2.4 Simulation output

2.2.4.1 Visualization

Simulation data can be visualized in various ways. The method of visualization can be configured in the `Analysis` element. `Gnuplotter` is the most versatile tool for visualization of 2D simulations. It is based on the graphing utility Gnuplot. This

Table 2.1: **Output files**. Description of common output and result files.

File	Description
model.xml	XML model generated by GUI upon model execution.
model.xml.out	Standard output from simulator (as in Output panel).
model.xml.err	Error message generated by the simulator.
[Title][Time].xml.gz	Compressed XML model with simulation state, created if and only if checkpointing enabled (Time/SaveInterval).
logger_[Symbols].log	ASCII text output from the Analysis/Logger plugin.
cells_[Time].png /jpg/gif/pdf	Plot generated by the Analysis/Gnuplotter plugin.
gnuplot_error.log	Gnuplot error messages (if any).

analysis plugin can visualize both cells and PDE layers, provides customizable color scales, and can display output to screen (wxt, aqua, x11, win) or write to file in a number of image formats (png, jpg, pdf, svg).

For 3D simulations, the TIFFPlotter provides writing image stacks that may contain 2D-5D data in the format [X, Y, Z, time, channel] that can be read by external image analysis software such as Fiji or ImageJ. Alternatively, 3D simulation data can be written to VTK format using VTKPlotter for post-hoc visualization with ParaView.

The frequency of visualization is set by the interval attribute of the particular Analysis element.

2.2.4.2 Logging and analysis

Simulation data can be exported to log files, either as raw data or in processed form. The Logger plugin writes cell property values or PDE values, optionally reduced after summing or averaging. The SpaceTimeLogger provides a way to write values of a linear PDE layer or a slice of a 2D/3D PDE layer to construct space-time plots. The HistogramLogger constructs and outputs frequency distributions of cell properties in a population.

The result files are standard tab-delimited text files that can be imported and processed by external statistical software. Additionally, these logging tools include a Plot function to visualize the data.

The frequency of data output is set by the interval attribute of the particular Analysis element.

2.2.4.3 Output folder

Simulation data is written to a folder [Title]_[JobID] within the simulation folder configured in Settings/Local. Before execution, the model XML file (i.e. model.xml) and dependent files (e.g. TIFF images) are written/copied to this folder. During execution, simulation output is written to the same folder. The contents of the output folder can be browsed by selecting the job in the JobQueue panel. Table 2.1 gives an overview of the files in the output folder.

Table 2.2: **SBML import**. Translation of SBML concepts (red) into Morpheus model description language (blue).

SBML	Morpheus MDL	Comment
Species	Property	Units are discarded
Parameter	Property / Constant	Depends on constant attribute
InitialAssignment	InitProperty	
Functions		Inlined in RateRule
AssignmentRule	Equation	
RateRule	DiffEqn	
AlgebraicRule	not supported	
Reaction		Converted to RateRule
Event	Event	Delays are not supported

2.2.5 Restoring and checkpointing

Models in the job archive can be restored by opening the file: `model.xml`. This opens the initial state of the simulation model as a new document.

When using checkpointing (`Time/SaveInterval`), the complete simulation state is saved as a compressed XML file (`[Title][Time].xml.gz`) at the specified interval. These files can be restored in the same way, however, in this case, a new document is opened containing the complete simulation state at time `[Time]`.

2.2.6 SBML import

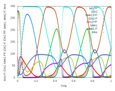

Morpheus supports importing SBML models for intracellular dynamics. SBML (Systems Biology Markup Language) is a standard for describing models of biochemical pathways. SBML models can be generated using SBML simulators such as Copasi or downloaded from public repositories such as the BioModels database.

Upon importing an SBML model, the SBML model is converted into systems of ordinary differential equations for intracellular dynamics in Morpheus model description language (MDL). Some SBML constructs can be translated in a one-to-one fashion into Morpheus MDL while others require a more elaborate conversion (see table 2.2).

In general, SBML models are imported in the following way (SBML constructs in red and Morpheus MDL constructs in blue):

1. A new non-spatial single-cell Morpheus model is created. This model specifies a single lattice site and a population of 1 cell. A `CellType` is created to contain the system of ordinary differential equations.

2. Matching concepts such as `Species`, `Events`, `InitialAssignments`, etc. are translated from SBML into the Morpheus MDL concepts of `Property`, `Constants`, `Events`, `InitProperty`, etc..

3. `Reactions` in the SBML model are, together with the associated `KineticLaws` and `Functions`, converted into SBML `RateRules`. These `RateRules` are

subsequently assembled into a System of differential equations (DiffEqn) such that a single differential equation describes the temporal evolution of a Species or Parameter.

4. Unlike in SBML, the symbolic identifier of a Property or a Constant must be unique in Morpheus MDL. Therefore, if necessary, Reaction parameters are renamed upon import by appending the sequential reaction number.

5. Simulation details are not specified in SBML models but are required for simulation. Therefore, default values are set during the import process. Simulation time is assumed to run from $0...1$ by setting Time/StartTime="0" and Time/StopTime="1". The System solver is set to Runge-Kutta (RK4) by setting solver ="runge-kutta" and its time-step="0.01".

6. Finally, simulation output is preconfigured with a graphical visualization of the time course data of all species using Analysis/Logger/plot.

Note that not all SBML concepts can be converted into Morpheus MDL. In particular, multiple compartments are not supported, and conversion of delay equations is not possible (since Morpheus only supports delay differential equations with constant delays, see section 3.2.3). Furthermore, note that units and dimensions are discarded upon import.

After importing an SBML model into Morpheus, it can be extended into a spatial, multicellular or multiscale model. Alternatively, it can be copied as a module into an existing model using the Clipboard (see section 2.1.7).

2.3 Parameter sweeps

The GUI provides a convenient interface for batch processing by generating multiple jobs with different parameter sets. All parameters (Attributes and Expressions) can be selected for parameter sweep, using the context menu item ParamSweep. Upon selection, the parameter appears in the ParamSweep view (fig. 2.3). This is opened when selecting the ParamSweep element in the Documents panel. This shows the XML path of the parameter and its type. The range of values for batch processing can be set be editing the values in the third column. Values can be given explicitly as semicolon-separated lists or using the list expansion syntax as shown in table 2.3.

2.3.1 Multiple parameters

By default, parameter sets will be combined in a combinatorial fashion, such that a job is created for each combination of parameter values. Note that the number of simulation jobs can get prohibitively large for multiple parameters. The number of jobs is calculated for the current configuration and displayed above the parameters.

To have multiple parameters set simultaneously changing sets, a parameter can be dragged on another to couple them in a pairwise fashion. Note that these parameter sets should be of the same length, otherwise, the lowest number of parameter values is used for the paired set.

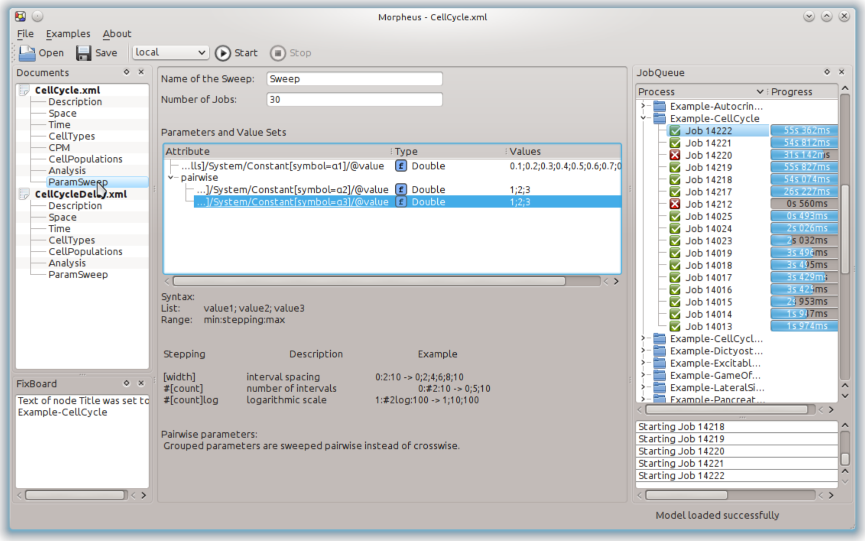

Figure 2.3: **Parameter sweep view**. This view is opened when selecting the ParamSweep element in the Documents panel.

Table 2.3: **Syntax for list expansion** in parameter sweep view.

		Syntax	Examples	Result
**List**		x;x;x	1.0;1.5;3.0	1.0;1.5;3.0
			10 10 10;20 20 20	10 10 10;20 20 20
			square;hexagonal	square;hexagonal
**Range**	Increment	x:x:x	0:2:10	0;2;6;8;10
			0.5:1:3	0.5;1.5;2.5
	Intervals	x:#x:x	0:#2:10	0;5;10
			0:#4:1.0	0;0.25;0.5;0.75;1.0
	Logarithmic	x:#xlog:x	1:#2log:100	1;10;100

2.3.2 Simulation and results

Parameter sweeps are executed from the ParamSweep panel using the Start button in the toolbar. Note that the ParamSweep panel must be opened in order to start a parameter sweep. Upon execution, a folder `[Name of Sweep]` is created that contains a file `sweep_summary.txt` and subfolders for each simulation job. The file contains the folder names for the individual jobs and their parameter sets. and can be used for post-hoc analysis.

2.3.3 Restoring parameter sweeps

Settings of previous parameter sweeps can be restored from the JobQueue provided that the selected model defined the same parameters and symbols.

3

Model formalisms

Morpheus supports the simulation of discrete cellular Potts models as well as continuous ordinary differential equations (ODEs) and reaction-diffusion systems. These core model formalisms are implemented in a modular way such that they can be flexibly integrated into multiscale models. Moreover, the modularity enables them to be combined into auxiliary formalisms such as finite state machines, cellular automata, coupled ODE lattices or gradient-based models.

3.1 The `System` construct

The `System` is a mathematical construct in Morpheus MDL that plays a central role in modeling of temporal dynamics including ordinary differential equation, reaction-diffusion systems as well as rule-based models such as cellular automata.

A `System` is an environment for tightly coupled sets of (differential) equations. Rules or differential equations in a `System` environment are updated synchronously such that the states of variables at time t are calculated on the basis of the state of the variables at the previous time $t - \Delta t$. Algebraic loops within or between rules or differential equations are allowed (only) within a `System`. Therefore, recurrence equations (`Rule`) or tightly coupled differential equations (`DiffEqn`) can be modeled within this environment.

A `System` is associated with a numerical `solver` and a `time-step` (as explained in section 3.2.1). The `time-step` sets the integration time step used by the solver and should be set by the user on the basis of performance (large time-step) and accuracy (small time-step) considerations.

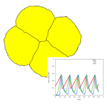

In addition, the `System` has an optional attribute `time-scaling` (default $= 1.0$) which scales the time within a System relative to the global time. This automatically scales the `System` `time-step` such that the numerical accuracy is preserved. The `time-scaling` attribute provides a convenient way to scale the dynamics of sub-models to each other.

3.2 Differential equations

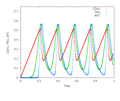

A number of numerical solvers are available for (a subset of) ordinary, stochastic, delay and partial differential equations to solve initial value problems (Cauchy

Table 3.1: **Numerical solvers** for ordinary and stochastic differential equations.

Method	Numerical scheme
**Euler**	$y_{n+1} = y_n + \Delta t f(t_n, y_n)$
**Heun (midpoint)**	$y_{n+1} = y_n + \frac{1}{2}\Delta t f(t_n, y_n) + f(t_{n+1}, \tilde{y}_{n+1})$ where $\tilde{y}_{n+1} = y_n + \Delta t f(t_n, y_n)$
**Runge-Kutta (RK4)**	$y_{n+1} = y_n + \frac{1}{6}\Delta t(k_1 + 2k_2 + 2k_3 + k_4)$ where $k_1 = f(t_n, y_n)$, $k_2 = f(t_n + \frac{1}{2}\Delta t, y_n + \frac{1}{2}\Delta t k_1)$, $k_3 = f(t_n + \frac{1}{2}\Delta t, y_n + \frac{1}{2}\Delta t k_2)$, $k_4 = f(t_n + \Delta t, y_n + \Delta t k_3)$
**Euler-Maruyama**	$y_{n+1} = y_n + \Delta t f(t_n, y_n) + \sqrt{\Delta t}\Delta W_n$
**Heun-Maruyama**	$y_{n+1} = y_n + \frac{1}{2}\Delta t f(t_n, y_n) + \sqrt{\frac{1}{2}\Delta t}\Delta W_n + f(t_{n+1}, \tilde{y}_{n+1})$ where $\tilde{y}_{n+1} = y_n + \Delta t f(t_n, y_n) + \sqrt{\frac{1}{2}\Delta t}\Delta W_n$

problems) of the form $\frac{dy}{dt} = f(t, y(t))$ together with the initial condition $y(t_0) = y_0$. Differential equations (`DiffEqn`) must be specified inside a `System` element that provides an environment for synchronous updating of tightly coupled updated differential equations.

3.2.1 Finite difference solvers

Morpheus implements finite difference methods, Euler, Heun and Runge-Kutta (see table 3.1). The method to be used for a set of differential equations is specified in `System/solver`. All solvers have a fixed time-step that must be specified by the user in `System/time-step`.

Note that forward solver methods are not suitable for solving stiff systems and may require small time-steps to guarantee stability and sufficient accuracy. All solvers use fixed time stepping; adaptive time stepping solvers are not yet implemented.

3.2.2 Stochastic differential equations

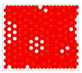

For stochastic differential equations, the Euler-Maruyama or Heun-Maruyama method is used (see table 3.1). These methods scale the noise amplitude to the time-step h that is used. Morpheus automatically switches to Maruyama solvers

when a DiffEqn contains a normally distributed random number, i.e. rand_-norm([mean],[stdev]).

Note that other random number distributions, e.g. uniform or gamma distributions, are not allowed in this context. Moreover, stochastic differential equations cannot be solved using the Runge-Kutta method.

3.2.3 Delay differential equations

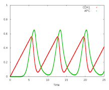

Morpheus supports delay differential equations through the use of a property with a delay, a DelayProperty. This property has an attribute delay that specifies the length of history, i.e. the lag between assignment of a value and the time at which it is returned.

Note that only constant time delays are supported. Further note that the delay must be a multiple of the solver time-step*time-scaling. Also note that a delay smaller than this time-step has no effect.

3.2.4 Initial condition

Initial conditions for cell-bound variables, i.e. Celltype/Property can be specified in various ways, depending on whether the initial condition should be specified in a homogeneous or heterogeneous way or to set an initial conditions in a cell-specific fashion.

3.2.4.1 Homogeneous population

The value specified in Celltype/Property/value serves as an initial condition for the whole population of this Celltype. Therefore, this method defines a cell population that is homogeneous with respect to this variable.

3.2.4.2 Heterogeneous population

A heterogeneous cell population in which initial conditions differ per cell may be configured using a mathematical expression in Population/InitProperty. This enables setting initial conditions according to cell ID (cell.id), cell position (cell.center.x) or using random distributions (e.g. rand_uni([min],[max])).

3.2.4.3 Cell-specific initial conditions

Initial conditions may be specified for each cell separately in Population/Cell/-PropertyData, and likewise for vector and delay properties. When using check-pointing, the state of each cell-bound variable is stored here.

3.3 Reaction-diffusion models

Solvers are available for initial boundary value problems on a one-, two- or three-dimensional domain of length L of the form $\frac{\partial y}{\partial t} = D\frac{\partial^2 y}{\partial x^2} + f(t, y(t))$ with initial

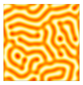
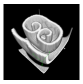

conditions $y(t_0, x) = y_0(x)$ and a set of boundary conditions such as $y(t, 0) = y(t, L) = 0$. Reaction-diffusion systems are solved using the sequential operator splitting method in which the original problem is split into two subproblems (the reaction and diffusion steps) that are solved sequentially, both for the same time-step. For the reaction step, one of the finite difference methods is used, as described above (section 3.2).

3.3.1 Diffusion

The diffusion equation is solved using the central difference method. The diffusion coefficient D is specified for each species in the reaction-diffusion systems in `Layer/Diffusion` (default: 0.0). The spatial discretization h is specified in `Space/Lattice/NodeLength` (default: 1.0).

During initialization, the numerical time-step for the reaction step is adopted by the diffusion problem. If necessary, the time-step is automatically adjusted in order to satisfy the Courant–Friedrichs–Lewy (CFL) condition, $2^d D \frac{\Delta t}{h^2} \leq 1$ where $d = 1, 2, 3$ is the number of dimensions.

3.3.2 Boundary conditions

Boundary conditions can be either `periodic` (default), `constant` (Dirichlet) or `noflux` (Neumann). These are specified in `Space/Lattice/BoundaryConditions` for each boundary separately (`x`, `-x`, `y`, `-y`, `z`, `-z`). Note that the type of boundary condition is shared for all spatial model components (although values may differ).

3.3.2.1 Constant boundary value

In case of constant boundaries, for each species in the reaction-diffusion system, a value for each boundary should be specified in `PDE/Layer/BoundaryValue` (default: 0.0).

3.3.2.2 Irregular domains

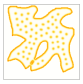

To solve reaction-diffusion systems in irregular domains, as often required in image-based systems biology, a shaped domain may be loaded from a TIFF image in `Space/Lattice/Domain/Image`. This TIFF must be in 8-bit format in which zero pixels are interpreted as background (outside domain), non-zero pixels are set as foreground (inside domain). Irregular domains can have `constant` or `noflux` boundaries.

3.3.3 Initial condition

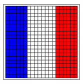

Initial conditions for each species in the reaction-diffusion system can be set using mathematical expressions in `PDE/Layer/Initial/InitPDEExpression`. This enables setting up random initial conditions or spatial gradients. By specifying symbols for space and lattice size using user-defined symbols (see table 4.1), heterogeneous initial conditions can be specified, independent of spatial discretization.

Table 3.2: **Cellular Potts model parameters** and their location in the model description language.

	Symbol	Description	Morpheus MDL
**Metropolis kinetics**			`CPM/MetropolisKinetics`
	T	Temperature	`temperature`
	Y	Yield	`yield`
	$\mathcal{N}$	Neighborhood	`Neighborhood`
		Sampling / stepper algorithm	`stepper`
**Interaction energy**			`CPM/Interaction`
	J	Interface energy	`Contact`
**Shape constraints**			`CellTypes/CellType`
	V_t	Target volume	`VolumeConstraint/target`
	λ_V	Strength of volume constraint	`VolumeConstraint/strength`
	P_t	Target perimeter	`SurfaceConstraint/target`
	λ_P	Strength of perimeter constraint	`SurfaceConstraint/strength`
**Non-Hamiltonian**			`CellType/CellType`
	μ	Chemotactic sensitivity	`Chemotaxis/strength`

3.4 Cellular Potts model

The cellular Potts model (CPM) is a cell-based time-discrete method that represents individual cell shapes as lattice domains and models cell motility in terms of energy minimization using modified Metropolis kinetics. Table 3.2 provides an overview of how cellular Potts model parameters are encoded in Morpheus MDL.

3.4.1 Modified Metropolis kinetics

A CPM defines cell shape and motility constraints in terms of energetical constraints, described in a Hamiltonian. Motility arises from updating the lattice configuration according to energy minimization, based on a modified Metropolis kinetics, with the following steps.

First, a lattice site $\boldsymbol{x}$ (target site) is chosen at random with uniform distribution (see 3.4.2). Second, a lattice site $\boldsymbol{x}'$ (trial site) is chosen from the neighborhood $\mathcal{N}_{\boldsymbol{x}}$, with uniform distribution (see 3.4.3). Then, the change in free energy ΔH is calculated for the case if the state σ at the trial site $\boldsymbol{x}'$ would be copied to the target site $\boldsymbol{x}$. Finally, whether or not this transition is accepted depends on ΔH according to a Boltzmann probability:

$$P(\sigma_{\boldsymbol{x}'} \rightarrow \sigma_{\boldsymbol{x}}) = \begin{cases} 1 & \text{if } \Delta H + Y < 0 \\ e^{-(\Delta H + Y)/T} & \text{otherwise} \end{cases}$$

Here, T (for 'temperature') modulates the probability of unfavorable updates to be accepted, and represents local protrusions/retractions of the cell membrane. The parameter Y (for 'yield') accounts for dissipative effects and represents for example cytoskeletal resistance to membrane fluctuations.

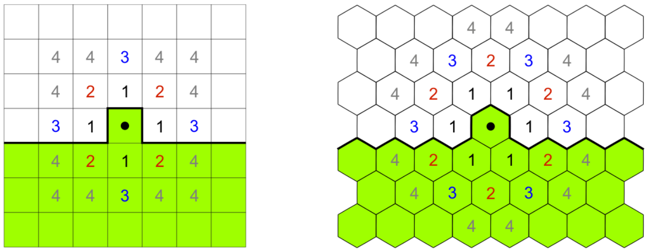

Figure 3.1: Neighborhood `Order`. Neighborhood of a lattice site (with dot) numbered according to the minimum neighborhood order in which they are included, for square (left) and hexagonal (right) lattices.

The parameters for the modified Metropolis kinetics are specified in `CPM/MetropolisKinetics`.

3.4.2 Stepper

In the standard `random` stepper algorithm, the target site x is selected at random, while the trial site x' is selected from its local neighborhood. In certain cases, this can be highly inefficient. In sparsely populated lattices, for instance, there is a high likelihood of selecting sites with identical states that cannot change the configuration. Selecting such sites is therefore redundant.

To prevent such meaningless updates, Morpheus provides the `edgelist` stepper. This sampling algorithm tracks all lattice sites that can potentially lead to a change in configuration and selects the target site x from this list with uniform random distribution. This can yield major improvements in computational efficiency without affecting model results.

The stepper algorithm can be selected in `CPM/MetropolisKinetics/stepper` (default: `edgelist`).

3.4.3 Neighborhood

The size of the `Neighborhood` $\mathcal{N}$ can be specified using either `Distance` or `Order`. The `Distance` specifies the maximum Euclidean distance within which lattice sites are considered neighboring sites. The `Order` uses a labeling scheme to identify the neighboring sites. These labels are integer values that alternate between axial (odd numbers) and radial (even numbers) neighborhoods as shown in figure 3.1.

3.4.4 Monte Carlo Step (MCS) and time scaling

Within the CPM, a Monte Carlo step (MCS) is often interpreted as a discrete unit of time. A single Monte Carlo step is defined as the number of random sampled updates equal to the number of lattice sites. That is within one MCS, on average, each lattice site has been sampled for an update.

The duration of a single MCS is scaled to the global simulation time as specified in `CPM/MCSDuration` (default: 1.0).

3.4.5 Cell shape, interaction and motility

3.4.5.1 Hamiltonian

Each cell occupies a set of lattice sites $\boldsymbol{x}$ with its cell index $\sigma > 0$, whereas $\sigma = 0$ refers to the medium. Changes in the spatial configuration of cells on the lattice are governed by a Hamiltonian H that describes the free energy of the lattice configuration. In its simplest form, ignoring intercellular interaction energies, $H = \sum_{\sigma>0} \lambda_V (v_\sigma - V_t)^2$ where v_σ is the actual volume (i.e. number of lattice sites) of cell σ and V_t is the target volume. Deviations from the target volume increase the free energy H according to the elasticity parameter λ_V.

Additionally, a constraint on the perimeter of the cell is often used, $H = \sum_{\sigma>0} \left[\lambda_V (v_\sigma - V_t)^2 + \lambda_P (p_\sigma - P_t)^2\right]$, where p_σ is the actual perimeter (i.e. number of interfaces between lattice sites) of cell σ and P_t is the target perimeter with λ_P representing the elasticity parameter. The targets and scalars are written here as constants for simplicity, but can be cell-specific and time-dependent, i.e. $V_{t,\sigma}(t)$, by linking them to cell-bound variables or functions.

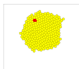

Hamiltonian terms for CPM models that constrain cell shape (`VolumeConstraint`, `ShapeConstraint`, `LengthConstraint`) can be specified in `CellTypes/CellType`.

3.4.5.2 Interaction energies

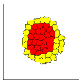

The CPM has been originally developed to study the effects of differential adhesion on cell sorting. Adhesion can be modeled using interaction energies that define a free energy penalty per interface of contact between cells. Differential cell adhesion can be modeled by specifying different interaction energies for contacts between different cells σ of different cell types τ. This extends the Hamiltonian with the term $H = \sum_{interfaces\, i,j} J\left[\tau(\sigma_i), \tau(\sigma_j)\right](1 - \delta_{\sigma_i \sigma_j})$ where J is a matrix of interaction energies between different cell types $\tau(\sigma_i)$ and σ_i is the cell at lattice site i. The Kronecker delta $\delta_{\sigma_i \sigma_j} = \{1, \sigma_i = \sigma_j; 0, \sigma_i \neq \sigma_j\}$ ensures only interfaces *between* cells are taken into account.

The matrix of interaction energies between cell types can be specified in `CPM/-Interaction`. These energies are normalized by the number of neighbors such that the interaction energies are automatically rescaled when the lattice structure is changed.

Interaction energies can be altered based on the state of a cell property that represents e.g. cadherin expression (`AddonAdhesion`) or on the combination of properties between neighboring cells to represent binding between heterophilic (`HeterophilicAdhesion`) or homophilic adhesion molecules (`HomophilicAdhesion`). These plugins can be specified in `CPM/Interaction/Contact`.

3.4.5.3 Kinetic terms

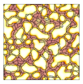

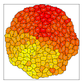

The classical CPM has been extended by non-Hamiltonian terms. Since these terms directly affect the change in energy ΔH and may change the energy of the system, they are called kinetic terms. A widely used kinetic term biases motility of a cell σ in the direction of a local concentration gradient of a species w in a reaction-diffusion model in order to represent chemotactic migration: $\Delta H = \sum_{\sigma>0} \mu(w_{\boldsymbol{x}'} - w_{\boldsymbol{x}})$.

Kinetic terms that bias motility (`Chemotaxis`, `Haptotaxis`, `DirectedMotion`, `Persistence`) can be specified in `CellTypes/CellType`.

3.4.5.4 Event-based terms

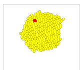

Other CPM extensions are based on the cell state at the end of a Monte Carlo step and are evaluated only once per Monte Carlo step, instead of every update trial. This includes extensions that model cell division (`Proliferation`) and cell death (`Apoptosis`). `Proliferation` triggers the division of a cell into two daughter cells, based on a condition. `Apoptosis` triggers the immediate removal of a cell (`lysis`) or setting its target volume to zero (`shrinking`) and removing the cell from the population only after it does not occupy any lattice sites.

3.4.5.5 Update-preventing terms

Some CPM extensions act to prevent updates altogether, based on some criterion. These include the `Freezer` that disables all updates for a particular cell, based on a user-specified condition. It also includes the `ConnectivityConstraint` that ensures the cell is simply connected by preventing updates that would break this topological constraint.

3.4.6 Initial condition of spatial configuration

Simulations of cellular Potts models require the specification of at least one population of cells in `CellPopulations/Population`. By default, the specified number of cells is distributed randomly in space using a uniform distribution, each cell occupying a single lattice site.

Spatially structured initial populations can be configured using `InitRectangle` and `InitCircle`. These initializers attempt to fit the specified number of cells in a regular fashion, with each cell occupying a single lattice site. Note that artefacts may occur due to spatial discretization.

Initializing a population of cells with geometrical shapes can be configured using the `CellObjects` initializer. This allows for the specification of spheres, cylinders, boxes and ellipsoids that can be arranged along the orthogonal axes.

Cell populations can also be initialized from images using the `TIFFReader` initializer. This provides an interface to configure models from microscopy images. TIFF images may be in 8-, 16-, and 32-bit format and may contain multiple z-slices (image stacks). By convention, all pixels sharing a particular integer value will be added to the same cell. The option `keep_id` ensures that this value is used as a cell ID internally.

3.5 Auxiliary model formalisms

The modular design of the formalisms allows a number of auxiliary formalisms to be constructed. For instance:

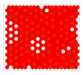

Coupled ODE Lattice Coupled ordinary differential equation lattice models can be used to represent a regularly structured tissue in which each cell is represented by an intracellular ODE model and communicates with its neighboring cells.

Coupled ODE lattice models can be constructed by configuring a lattice of cells, each occupying a single lattice site using `InitRectangle`. A `CellType` with a `System` of `DiffEqn` describing the intracellular dynamics. Intercellular communication is modeled using a `NeighborReporter` that reports the (weighted) average of the properties of directly adjacent neighbor cells.

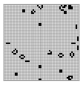

Cellular Automata (CA) Cellular automata are a widely used discrete-time, discrete-space, discrete-variable formalism to study the emergence of macroscopic behavior from microscopic local rules.

CA models can be constructed by configuring a lattice of cells, each occupying a single lattice site using `InitRectangle`. A `CellType` with a `System` of synchronously updated `Rules` describes the state transitions, based on the states of cells in the local neighborhood, reported using a `NeighborReporter`.

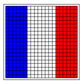

Gradient based models Gradient-based models can be used to describe patterning of tissues under influence of a morphogen gradient, such as Wolpert's classical French flag model.

Gradient-based models are built using a non-diffusive `PDE Layer`, initialized by a mathematical expression using an `InitPDEExpression`. A regular lattice of cells is configured by an `InitRectangle`, in which each cell measures the morphogen concentration at its location using a `PDEReporter`, based on which an `Equation` defines the cellular identity.

4

Model description language

Morpheus simulation models are specified in a custom domain-specific model description language (MDL). The XML-based language uses biological and mathematical terminology to declaratively describe multiscale multicellular simulation models. It is composed of human-readable tags to represent the components of biological processes (table 4.2) and a number of mathematical constructs to define their dynamics and relations (table 4.3).

Morpheus simulation models are fully specified by single model description files. These include the definition, configuration and and parameterization of (sub)models as well as the specification how these (sub)models are interlinked. Details on the numerical simulations are also stored in the model description file such as the simulation time, spatial discretization, initial conditions and the configuration of visualization and data output. For checkpointing, the complete state of a simulation during execution can be stored in the same file format. The fact that the complete simulation model, including the description of its dynamics, is encapsulated in single files, render them suitable for archiving as well as model exchange between users.

4.1 Declarative

The Morpheus model description language (MDL) separates modeling from implementation by allowing the description of models in a declarative fashion. Models describe *what* processes are to be simulated rather than *how* this should be accomplished. This distinguishes declarative languages such as the Morpheus MDL from imperative programming language such as C++ or Python that focus on the description of algorithmic control flow.

4.2 Domain-specific

The MDL uses a domain-specific markup language using vocabulary that is derived from the application domain of multiscale and multicellular systems biology. On the one hand, it uses concepts such as cell types and populations, and biological processes such as proliferation and chemotaxis (table 4.2). On the other hand, it defines a range of mathematical constructs such as constants, functions, equations

and systems of differential equations (table 4.3). This combination of biological and mathematical terms provides a powerful way to describe the relations and dynamics of biological processes.

4.3 Encapsulation

Models in Morpheus MDL describe both the model itself and specify details of its simulation including initial conditions (see table 4.2). During a simulation, the full simulation state, including the position and state of all cells, can be written in the same description language. In this way, a single model file contains the full specification of the model simulation. The encapsulation in a single file significantly simplifies archiving, checkpointing and restoring simulation models as well as the exchange of models between users.

4.4 Two-tiered architecture

Morpheus MDL has a two-tier architecture (see figure 4.1). On the one hand, the XML is used to store information about the model components or sub-models in a hierarchically structured way. On the other hand, symbolic interdependencies represent the interactions and feedbacks between model components or sub-models. This combination provides a convenient way to express models of complex biological processes and allows automation of model integration (see chapter 5).

4.4.1 XML

The model description language is based on the eXtensible Markup Language (XML). This has a number of advantages: It stores information in a well-structured fashion that can be easily parsed and validated and it allows human-readable domain-specific terminology and can be extended in a straightforward fashion.

The main elements of the XML structure, as shown in table 4.2, are used to describe both the structure and parameterization of the model, and the details of its numerical simulation. The spatio-temporal aspects of the simulation are specified in the required `Space` and `Time` elements, the initial conditions or simulation state are described in `CellPopulations`, data output and visualization is configured in `Analysis`. The title and annotation of a model are added in the `Description` element.

The model itself is configured using the `CellTypes`, `CPM`, and `PDE` elements. The properties, behavior and dynamics of cells, including intracellular dynamics, are specified in the `CellTypes` element. The parameters of the cellular Potts model are configured in the `CPM` element and the reaction-diffusion models are defined and configured in the `PDE` element. These elements are all optional such that the various model formalisms can be used in isolation as well as in combination.

The XML represents this information in a hierarchical tree-like structure that reflects the structure of the modeled biological system. For instance, the main element `CellPopulations` can contain a `Population` that contains multiple `Cells`.

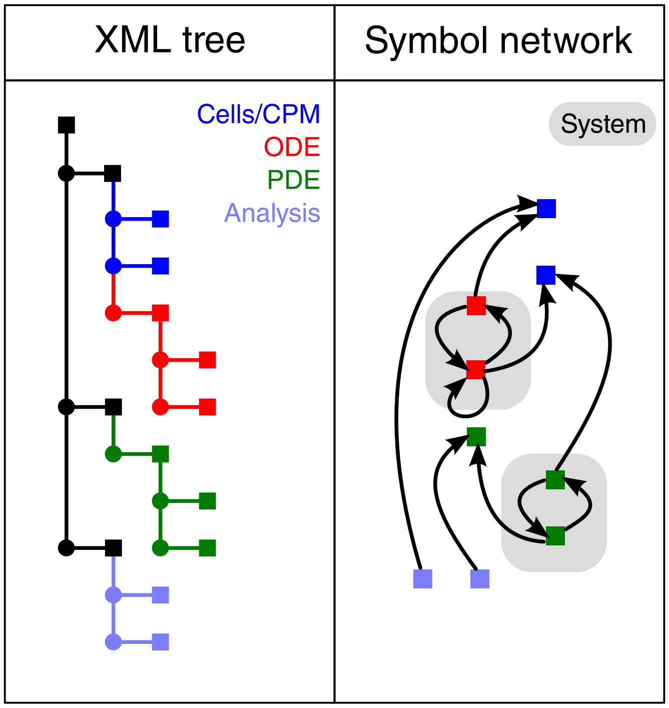

Figure 4.1: Schematic representation of the two-tier architecture. The hierarchical XML tree provides information on the structure of the model and its components (colored boxes). By defining and referring to symbolic identifiers, model components can be linked together in a network (arrows). `Systems` (rounded grey boxes) provide an environment for tightly-coupled differential equations in which self-references and circular dependencies between symbols are allowed.

Similarly, intracellular dynamics are modeled using a `Systems` of `DiffEqn` within a `CellType`, while the `PDE` describing extracellular dynamics is defined in its own element outside of `CellTypes`.

The XML structure is convenient to represent the hierarchy between the components of a model. However, it is not suitable to describe the network of interactions and feedbacks between these components, which is done using symbolic identifiers.

4.4.2 Symbols

Model components can be linked using symbolic identifiers. Symbolic identifiers and references establish interactions and feedbacks between (sub)models to represent the network-like complexity in biological processes (see fig. 4.1).

Symbolic identifiers, or symbols, can be specified to represent user-defined model variables such as cell-bound properties (`Property`) or concentrations of species in a reaction-diffusion model (`Layer`) (see table 4.1). Symbols can also

be specified for simulation-related constants and variables such as lattice size and current time of simulation (see table 4.1). Symbols can be used in mathematical expressions to define relations between model components. Additionally, symbols provide a convenient way to integrate different (sub)models by defining symbolic identifiers in one (sub)model and using them in another (sub)model.

4.5 Mathematical constructs

The model description language provides a number of mathematical constructs to define constants and variables and to express functions, equations and conditional events as well as the specification of tightly coupled systems of differential equations. An overview of the available constructs is given in table 4.3.

Mathematical expressions may be specified in terms of user-defined or predefined symbols (see table 4.1). Expressions are entered in plain text using standard infix notation. An overview of the available operators, functions and random number generators and their syntax is given in table 4.4.

Expressions are parsed during initialization using the fast math parser muparser (`muparser.beltoforion.de`). Muparser converts expressions to reverse Polish notation represented in byte code that is used to evaluate the mathematical expressions at run time.

4.6 XML schema

The rules, constraints and contents of the Morpheus model description language are laid down in an XML schema. The XML schema description (XSD) is embedded in the GUI and provides the information to edit, validate and repair model descriptions. Although the XML schema validates the XML structure, the GUI does not check the correctness of symbolic linking and mathematical expressions. Therefore, syntactically correct models may still result in simulation errors despite validation by XML schema.

Table 4.1: **User-defined and pre-defined symbols** in Morpheus model description language.

Context	Element	Type	Description
		**Simulation symbols**	
`Space`	`Lattice/Size`	vector	Size of lattice
	`SpaceSymbol`	vector	Current location
	`NodeLength`	double	Spatial discretization
`Time`	`StartTime`	double	Initial simulation time
	`StopTime`	double	Termination time of simulation
	`SaveInterval`	double	Interval between checkpointing
	`TimeSymbol`	double	Current time
		**Model symbols**	
Various	`Global`	double	Constant with global scope
	`Constant`	double	Constant with local scope
	`ConstantVector`	vector	Constant vector with local scope
	`Function`	double	Mathematical expression
`Celltype`	`Property`	double	Cell-bound variable
	`PropertyVector`	vector	Cell-bound variable vector
	`DelayProperty`	double	Cell-bound variable with delay
`CPM`	`MCSDuration`	double	Time of single Monte Carlo step
`PDE`	`Layer`	double	Reaction-diffusion species
		**Predefined symbols**	
cell	`cell.id`	integer	Unique cell index
	`cell.type`	integer	Cell type index
	`cell.volume`	integer	Number of lattice sites cell occupies
	`cell.surface`	integer	Number of lattice sites of cell boundary
	`cell.center`	vector	Center of mass of cell
	`cell.length`	double	Cell length of major axis
	`cell.orientation`	vector	Orientation of major axis
vectors	`[symbol].x/y/z`	double	Cartesian vector coordinates
	`[symbol].abs`	double	Magnitude of vector
	`[symbol].phi/theta`	double	Polar coordinates of vector

Table 4.2: **Model description language**. A summary of the main elements of the Morpheus description language and their most important sub-elements. Required (sub)elements are printed in boldface.

Element	Description	Sub-elements
**`Description`**	Sets the name (`Title`) of the model, used for naming the destination folder. May include model annotation (`Details`), used for human-readable annotation only.	**`Title`**<br>`Details`
**`Space`**	Sets the size, structure and boundary conditions of the lattice (`Lattice`). Optionally, sets a symbols for the lattice size and current location (`Lattice/Size/symbol` and `SpaceSymbol`).	**`Lattice`**<br>`SpaceSymbol`
**`Time`**	Set the duration of a simulation (`StartTime` and `StopTime`) defining the global time. Optionally, sets a symbol for current time (`TimeSymbol`). May specify the interval to save the simulation state (`SaveInterval`). May set a random seed for stochastic simulations (`RandomSeed`).	**`StartTime`**<br>**`StopTime`**<br>`TimeSymbol`<br>`SaveInterval`<br>`RandomSeed`
`CellTypes`<br>**`CellType`**	Allows multiple cell types to be defined. Each cell type (`CellType`) sets a name and type (i.e. biological or medium).<br>May define multiple properties (`Property`) for use in mathematical expressions (`Equation`, ...). May contain reporters for spatial mapping (`Reporter`). May define systems of ordinary differential equations (ODE) (`System/DiffEqn`). May specify a diversity of cellular behaviors (`Chemotaxis`, `Proliferation`, ...).	`Property`<br>`System`<br>`Constant`<br>`Function`<br>`Equation`<br>`Event`<br>`Reporter`<br>`Chemotaxis`<br>`Proliferation`<br>...
`CPM`	Sets the time-scale of a Monte Carlo step (`MCSDuration`), the parameters for the cellular Potts model (`MetropolisKinetics`) and the parameters of interactions between cells (`Interaction`).<br>Optionally, for constant boundary condition, sets a cell type at a boundary (`BoundaryValue`).	**`Interaction`**<br>**`MetropolisKin.`**<br>**`MCSDuration`**<br>`BoundaryValue`
`PDE`	Sets the symbol and diffusion coeffients for species (`Layer`) in a reaction-diffusion model for use in mathematical expressions (`Equation`, ...). May set a system of differential equations (`System/DiffEqn`) for reactions.	**`Layer`**<br>`Constant`<br>`System`<br>`Function`<br>`Equation`
`CellPopulations`<br>**`Population`**	Allows multiple populations to be defined. Each population sets a cell type and size (`Population`). May set initializers (e.g. `Initrectangle`). May explicitly specify multiple cells with properties and positions (`Cell`). When saving the simulation state, state of each cell is specified here.	`Cell`<br>`Initrectangle`<br>`TIFFReader`<br>...
`Analysis`	Sets the visualization and analysis tools. May contain various loggers and plotters (`Gnuplotter`, `Logger`). Executed at user-specified intervals.	`Gnuplotter`<br>`TIFFPlotter`<br>`Logger`<br>`HistogramLogger`

Table 4.3: **Mathematical constructs**. Overview of the mathematical constructs available in model description language (● = symbol definition, ○ = symbol reference).

Element	Description	Symbol graph
	**Containers**	
`Constant`	Constant value of type double with local scope, i.e. valid within the `CellType` or `System` it is defined in.	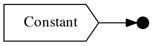
`Global`	Variable value of type double with global scope.	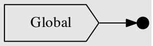
`Property`<br>`PropertyVector`<br>`DelayProperty`	Cell-bound variable. `Property` and `DelayProperty` are of type double. `DelayProperty` has attribute `delay` to set the lag between assignment and return of value. `PropertyVector` defines Euclidean vector in space delimited format "x y z".	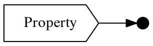
`Layer`	`PDE` model variable, i.e. species in reaction-diffusion system. Diffusivity of a `Layer` is specified in attribute `diffusion`.	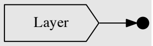
	**Expressions**	
`Function`	Mathematical expression. Computes a value (double) for the output symbol it defines, but does not assign it to a variable. Updated whenever when output symbol is referenced. May not contain algebraic loop.	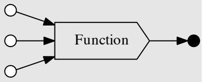
`Equation`	Mathematical expression. Computes a value (double) and assigns it to the variable it references. Updates are scheduled depending on its symbol dependencies. May not contain algebraic loop.	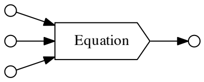
`Rule`	Mathematical expression that defines a (recurrence) equation for use in environments such as `System` and `Event`. Scheduled according to `System/time-step`. May contain algebraic loop and self-references.	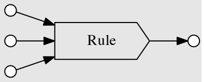
`DiffEqn`	Mathematical expression that defines a differential equation. Only allowed in `System` environment. May contain algebraic loop and self-references.	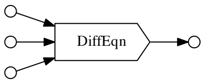
	**Reporters**	
`Reporter`<br>`NeighborsReporter`<br>`PDEReporter`<br>`...`	Explicit data mappings. Computes a statistic (average, mean, etc.) of the input data and assigns this to the output symbol. Updates are scheduled depending on its symbol dependencies.	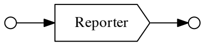
	**Environments**	
`System`	Environment for tightly coupled sets of differential equations and rules that are synchronously updated (see section 3.1). Scheduled according to user-specified `System time-step` and `time-scaling`.	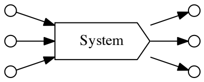
`Event`	Environment for conditional or timed events. Triggered periodically or, if `Condition` is specified, whenever the condition changes from false to true. Updates are scheduled according to `time-step` if specified or depending on its symbol dependencies otherwise.	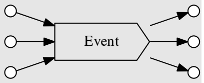

Table 4.4: **Operators and predefined functions** available in mathematical expressions.

Class	Description	Syntax
**Operators**	Addition	`+`
	Subtraction	`-`
	Multiplication	`*`
	Division	`/`
	Power	`^`
**Logical operators**	Logical and	`and`
	Logical or	`or`
	Exclusive or	`xor`
**Comparison**	Equal	`==`
	Not equal	`!=`
	Smaller	`<` or `<`
	Greater	`>` or `>`
	Smaller or equal	`<=` or `<=`
	Greater or equal	`>=` or `>=`
**Functions**	Sine	`sin(...)`
	Cosine	`cos(...)`
	Tangens	`tan(...)`
	Arc sine	`asin(...)`
	Arc cosine	`acos(...)`
	Arc tangens	`atan(...)`
	Hyperbolic sine	`sinh(...)`
	Hyperbolic cosine	`cosh(...)`
	Hyperbolic tangens	`tanh(...)`
	Arc hyperbolic sine	`asinh(...)`
	Arc hyperbolic cosine	`acosh(...)`
	Arc hyperbolic tangens	`atanh(...)`
	Logarithm base 2	`log2(...)`
	Logarithm base 10	`log(...)`
	Natural logarithm	`ln(...)`
	Exponent	`exp(...)`
	Power	`pow([base], [exponent])`
	Square root	`sqrt(...)`
	Sign, -1 if $x<0$, 1 if $x>0$	`sign(...)`
	Round nearest integer	`rint(...)`
	Absolute	`abs(...)`
	Minimum of arguments	`min(..., ..., ...)`
	Maximum of arguments	`max(..., ..., ...)`
	Sum of arguments	`sum(..., ..., ...)`
	Average of arguments	`avg(..., ..., ...)`
	Modulus, remainder	`mod([numer], [denom])`
**Random number**	Uniform distribution	`rand_uni([min], [max])`
	Normal distribution	`rand_norm([mean], [stdev])`
	Gamma distribution	`rand_gamma([shape], [scale])`
	Boolean (0 or 1)	`rand_bool()`
**Condition**	Conditional statement	`if([condition], [then], [else])`

5

Model integration

Morpheus supports the integration of time-discrete cell-based models with time-continuous models for intra- and extracellular dynamics. In particular, cellular Potts models (CPM) can be linked to ordinary, stochastic or delay differential equations as well as to reaction-diffusion (PDE) models. The Morpheus model description language facilitates the specification of links between (sub)models with the help of symbolic identifiers (see chapter 4).

For the user, a link between sub-models is established by simply defining a symbol in one sub-model and using it as an input in another sub-model, providing a convenient way to construct and explore complex multiscale biological systems using integrative models. During simulation, Morpheus makes the data accessible between sub-models and, if necessary, mapping or transforming it to make it suitable for the target sub-model. Moreover, the updates of the various sub-models are appropriately scheduled, by determining the correct order and the frequency of updates, as to guarantee that up-to-date data is used in all computations. Both tasks are handled automatically as far as possible, based on user-specified time-steps and symbolic interdependencies.

5.1 Spatial mapping

Integration of spatial model formalisms, i.e. cell-based and reaction-diffusion models, requires that the data from one sub-model is accessible to the other sub-model. In Morpheus, data is not copied between sub-models, but is directly accessible through symbolic identifiers. Yet, the data must be accessed in a way that is appropriate for the model that uses the symbol. The model that uses a symbol determines the lattice sites for which the symbol is resolved, whereas the model that defined it determines the value of the symbol at those lattice sites.

Morpheus uses the convention that the spatial discretization in cell-based and reaction-diffusion models is identical. The lattice size (`Lattice/Size`) and the physical interpretation of the size of a lattice site (`Lattice/NodeLength`) are shared by both CPM and PDE models, which simplifies their integration. Therefore, in some cases, the mapping is trivial enough to be handled automatically. In other cases, a more complex mapping is required such as calculating a sum or average which requires the explicit instructions by the user using a `Reporter`.

5.1.1 Automated mapping

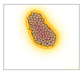

Mappings that present an unambiguous relationship are resolved automatically. For instance, when non-spatial constants and variables are used within a spatial context, the same value is used for all lattice sites over which is iterated. The use of symbols referring to cell-boundary `Properties` within reaction-diffusion (`PDE`) models linking `PDE` to `CPM` models can also be handled automatically. In that case, for each lattice site for which the `PDE Layer` is defined, the value of the cell property of the cell at that site is used.

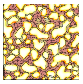

The reverse case, when `PDE` variables are used in `CPM` models, can be automatically mapped, only in certain cases. For instance, in the `Chemotaxis` extension, cell motility is biased by the gradient of concentration of a species in a reaction-diffusion model. Here, the difference in concentrations of the `PDE` variable at the target and trial site is automatically resolved (see 3.4.5.3).

5.1.2 Explicit mapping with `Reporters`

When the mapping between the two contexts is not unambiguous, a statistic is required to define how a symbol should be mapped. This requires the user to specify an appropriate way to transform the data from one (sub)model to the other (sub)model. Defining such an explicit mapping is done using a `Reporter`.

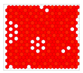

The `NeighborsReporter` calculates the (weighted) average or sum of the cell-bound `Property` in adjacent cells given by the input symbol and assigns this statistic to its output symbol.

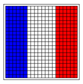

The `PDEReporter` maps `PDE` variables for use in cell-based models by reporting the average, sum, maximum or gradient of concentration of the input `PDE/Layer` at the lattice sites occupied by the cell or, optionally, its discretized center of mass.

5.2 Scheduling

Integration of time-continuous ODE and PDE models with time-discrete CPM models and various auxiliary mathematical constructs requires a careful scheduling of numerical updates. While the general simulation schedule is executed in fixed order, some processes must be scheduled according to the symbolic interdependencies in order to guarantee correctness of simulation results. The simulation schedule is computed during intializatoin and printed to the standard output (`model.xml.out`).

5.2.1 Simulation schedule

The general simulation cycle follows a fixed order in which temporal models are executed before the sequential processes and output processes, as shown in figure 5.1. The scheduling of sequential processes such as `Reporters` and `Equations` involves the determination of the order of updates as well as the setting of intervals in which the various model components should be computed.

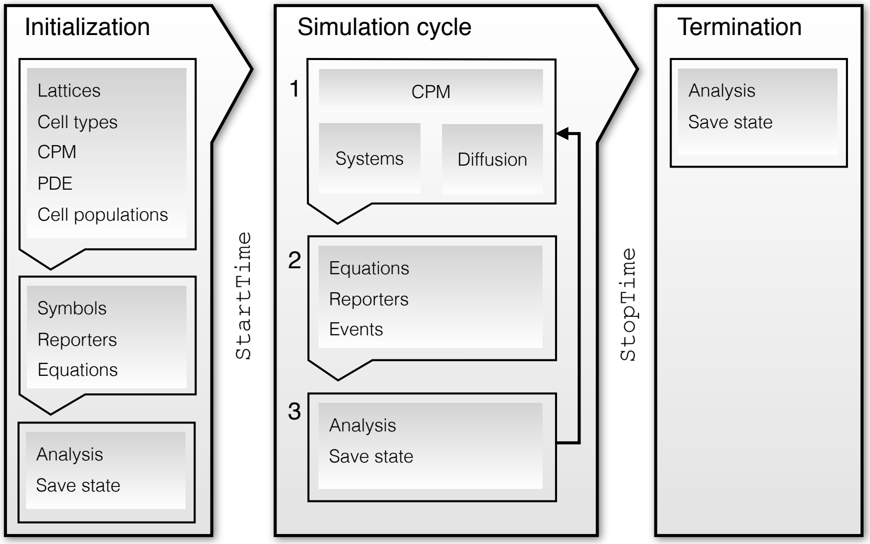

Figure 5.1: **Overview of the general simulation schedule**. Between initialization and termination, the simulation cycle is iteratively executed until time=`StopTime`. A single simulation cycle proceeds in three phases. First, the temporal models such as `CPM` , `Systems` and `Diffusion` are updated according to user-defined time-steps. Second, the sequential processes such as `Equations` and `Reporters` are updated in an order and interval that is determined by dependencies between symbols. Third, output is generated and the simulation state is written to file at user-defined intervals.

Initialization

First, the lattice is created and initialized, cell types are created, the CPM is initialized, and PDE layers are created and initialized (`PDEInitExpression`). Afterwards, populations of cells are created and put in the lattice randomly or according to a user-specified initializer (e.g. `InitRectangle`). Then, symbols are registered and set to initial values. Afterwards, `Reporters` and `Equations` are run once to initialize all remaining symbols. Finally, the analysis and visualization tools (`Analysis`) are run, and the simulation state is saved to file.

Simulation cycle

The simulation cycle is iterated from `StartTime` to `StopTime` in steps that advance according to the smallest time step (section 5.2.3). In each iteration, the processes that are scheduled for the current time step are executed in the following order.

1. **Temporal models**: The time-dependent models are executed. A single Monte Carlo Step is performed for the `CPM`. Afterwards, `Systems` and `Diffusion` are updated.

2. **Sequential processes**: Non-time-dependent models such as Equations, Reporters and Events are updated. Their order is computed from their symbolic interdependencies (section 5.2.2).

3. **Analysis**: Finally, visualization and data output is updated and the simulation state is saved to file.

Termination

At the end of simulation, visualization and analysis tools specified in Analysis tools can be executed once more and the simulation state is written to file.

5.2.2 Scheduling update order

The order of execution is independent of the order in which model components are specified in the model description file. Rather, updates of temporal processes (CPM, System, Diffusion) are scheduled according to the fixed schedule shown in figure 5.1 and updates of sequential processes such as Equations and Reporters are scheduled according to their symbolic interdependencies.

Sequential processes are ordered according to the dependencies in their input and output symbol based on the following rule:

- Before updating a process, its input symbols must all be updated.

This is achieved by scheduling all processes that have these symbols as an output before. Note that this is only possible if no algebraic loops or circular dependencies exist between these processes. Therefore, such loops are only allowed within the System environment.

The order of execution of data output and visualization in Analysis is arbitrary and does not affect the simulation itself. Therefore, these are executed in the order in which they are given in the model description file.

5.2.3 Update interval

The simulation cycle is iterated for the period from StartTime to StopTime, called the global simulation time. The number of iterations that are executed during this period depends on the temporal process with the smallest time step. During each iteration, only the processes that require updating are executed. The intervals at which each process requires updating are determined during initialization, based on user-specified time-steps or symbolic interdependencies.

The frequency in which CPM and Systems are updated is based on user-specified information. The update interval of CPM models is the duration of a single Monte Carlo step, specified in CPM/MCSDuration (see 3.4.4). The update interval of a System is determined by its time-step, divided by time-scaling (see 3.1). Similarly, the intervals at which Analysis processes are executed to do visualization and data analysis also depend on a user-specified interval.

The update intervals of other processes such as `Equations`, `Reporters`, `Events` and `Diffusion` are automatically determined by propagation of the intervals of their input and output symbols, according to the following rules:

- The process is updated as often as its output symbol(s) are used.
- The process is not updated more often than its input symbol(s) can change.

The former ensures that up-to-date data is used in all processes, while the latter improves computational performance by preventing redundant computations. Note that processes are not scheduled and computed if their output symbol(s) is (are) not used.

The interval of `Diffusion` is likewise determined by the interval of its output symbol, but it may iterate multiple times with smaller intervals as to satisfy the CFL stability criterion (see 3.3.1). `Functions` are not explicitly scheduled and do not have an update interval. Instead, they are updated whenever their output symbol is used, but at most once per minimal time step. User-specified intervals for `Analysis` processes smaller than the minimal time step are ignored, since the state of the simulation cannot change within this interval.

The final simulation schedule including the computed order of execution and update intervals is calculated at the end of initialization and printed to the standard output. This is written to the output file `model.xml.out` and is displayed in the output text box of results view in the GUI (see 2.2).

Table 5.1: **Example models** are available in the application and described on the website.

**Ordinary differential equations**				
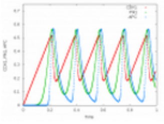	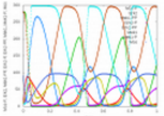	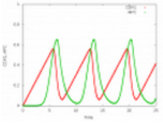	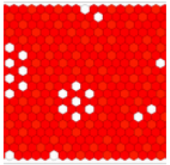	
**Reaction-diffusion systems**				
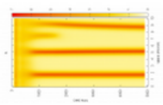	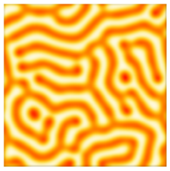	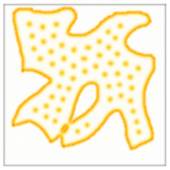	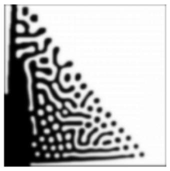	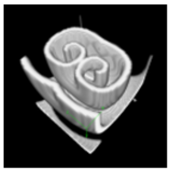
**Cellular Potts models**				
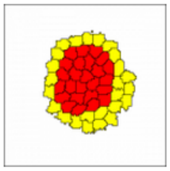	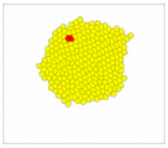	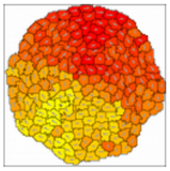	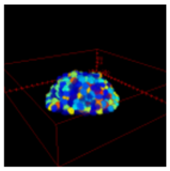	
**Multi-scale models**				
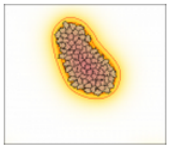	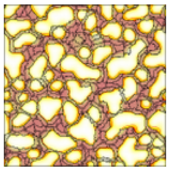	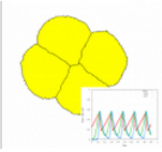	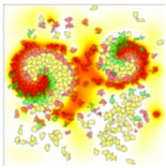	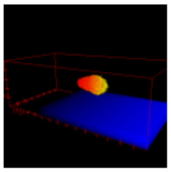
**Miscellaneous models**				
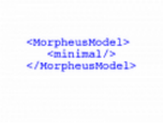	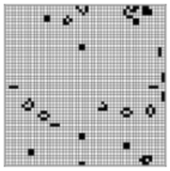	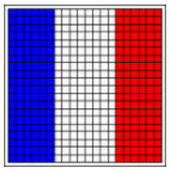		
